# Submental Approach Robotic‐Assisted Neck Dissection (SARAND) for Minimally Invasive Bilateral Neck Dissection: A Feasibility Cadaver Dissection Study

**DOI:** 10.1002/hed.70397

**Published:** 2026-07-14

**Authors:** Bartosz P. Wojtera, Karma Lambercy, Avinash Beharry, Ka Hei Suen, Markus Rheinwald, Christian Simon

**Affiliations:** ^1^ Department of Otolaryngology Head and Neck Surgery, Centre Hospitalier Universitaire Vaudois (CHUV), University of Lausanne (UNIL) Lausanne Switzerland; ^2^ Department of Head and Neck Surgery, Greater Poland Cancer Centre Poznan University of Medical Sciences (PUMS) Poznan Poland; ^3^ Department of Surgical Applications Engineering Intuitive Surgical Sunnyvale California USA

**Keywords:** bilateral neck dissection, da Vinci, endoscopic surgery, head and neck surgery, robotic surgery

## Abstract

**Background:**

Following promising initial results of the robotic‐assisted extended “Sistrunk” approach (RESA) that uses a submental incision to hypopharyngeal and laryngeal tumors, bypassing the base of tongue, we report its further development: submental approach robotic‐assisted neck dissection (SARAND), intended to enable bilateral neck dissections.

**Methods:**

Bilateral neck dissections (levels I–IV) were performed on three cadavers using both multiport and single‐port robotic platforms.

**Results:**

We demonstrate the feasibility of performing levels I–IV dissections through a single submental incision with both robotic platforms. A central working space was established over the hyoid bone, and boom rotation enabled optimal access to all neck levels from I to IV.

**Conclusion:**

SARAND appears to be a promising minimally invasive technique for both unilateral and bilateral neck dissection. It offers the potential to reduce neck morbidity compared to conventional approaches. Additionally, it potentially enables neck dissection and primary tumor excision via a median pharyngotomy during the same procedure.

## 1 Introduction

Transoral robotics surgery (TORS) has revolutionized the field of head and neck surgery, enabling minimally invasive approaches to upper aerodigestive tract tumors [1]. While TORS primarily focuses on oropharyngeal and supraglottic resections, its application has expanded to hypopharyngeal tumors, and both partial and total laryngectomy [2, 3, 4, 5, 6, 7]. TORS provides excellent oncological and functional outcomes, significantly outperforming other treatment modalities in selected patients [3, 5, 8, 9, 10, 11, 12, 13, 14, 15, 16, 17, 18].

While the transoral approach offers numerous advantages, significant challenges in exposing the hypopharynx and larynx often arise due to the individual anatomy of the base of tongue and hyoid bone or previous head and neck cancer (HNC) treatment [19, 20, 21]. The robotic‐assisted extended “Sistrunk” approach (RESA) was developed to overcome these limitations and provides patients with a minimally invasive and organ‐preserving surgical solution [22]. RESA provides adequate exposure, vascular control, a low complication rate, and satisfactory functional recovery, also in a salvage setting [23, 24].

Traditional open bilateral neck dissection is associated with significant morbidity, including a large visible scar and an increased risk for lymphedema [25]. Robotic‐assisted approaches—such as the retroauricular, trans‐axillary, and modified facelift—were developed to address these concerns [26, 27, 28]. Although feasible, these methods are time‐consuming and primarily designed for unilateral neck dissection, limiting their applicability in patients requiring bilateral procedure. Furthermore, they still involve substantial collateral tissue mobilization due to elevation of a skin‐platysma flap and the complete exposure of sternocleidomastoid muscle (SCM), which compromises lymphatic drainage of the skin and subcutaneous tissue [29]. These limitations have prompted the development of the submental approach, which offers the potential to reduce lymphedema, improve cosmetic outcomes, and preserve the greater auricular nerve to maintain skin sensation.

While not cleared for clinical use of these procedures to date, the da Vinci SP surgical system (Intuitive Surgical Inc.) enabled the development of additional remote‐access cervical approaches, including submental, presternal, clavicular/supraclavicular, and transcervical techniques, primarily in cadaveric studies in thyroid and upper aerodigestive tract surgery [30, 31, 32, 33]. These approaches demonstrated the feasibility of single‐port surgery within the confined cervical space while aiming to reduce flap dissection and improve cosmetic outcomes.

**FIGURE 1:**
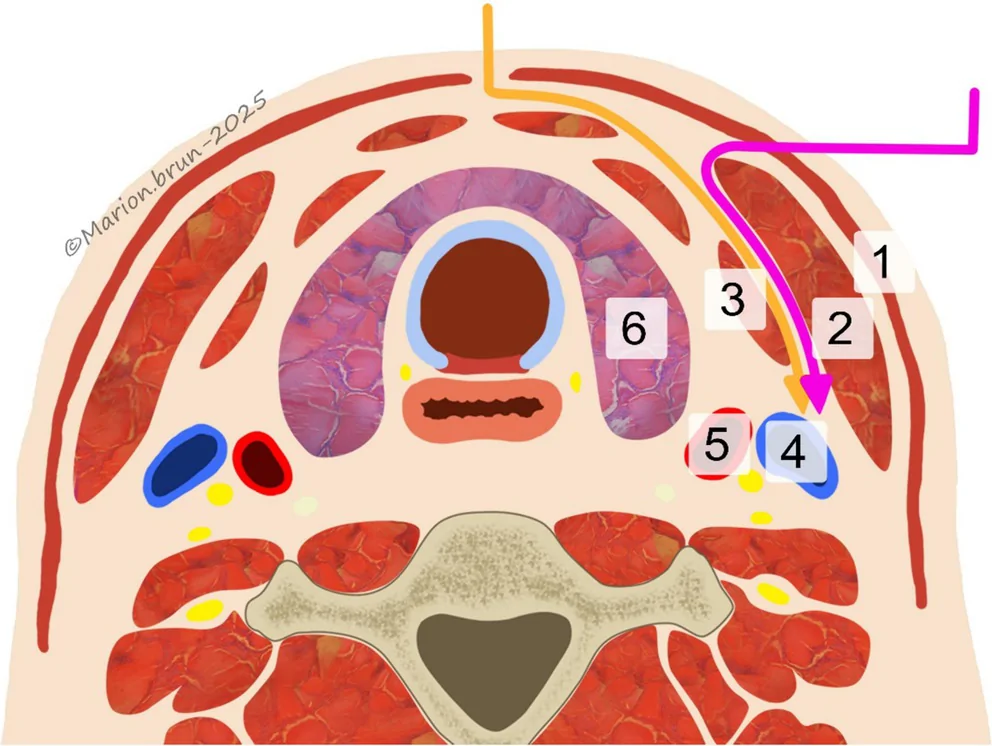
Difference between traditional approach to the neck lymphatics (pink) and the submental approach robotic‐assisted neck dissection (SARAND) (orange): During the traditional external approach the platysma (1) must be divided and elevated to expose the sternocleidomastoid muscle (2). Pre‐tracheal muscles are visible (3) and after following the investigating layer of the deep cervical fascia the carotid sheath with the internal jugular vein (4), and common carotid artery (5) become exposed. (6) Thyroid gland. During the SARAND a central working space is created over the hyoid bone. This is similar to the “Sistrunk” procedure, where this space is occupied by a thyroglossal duct cyst. After following the pre‐tracheal fascia and then the investing layer of the deep cervical fascia (orange) the carotid sheath can be exposed without violation of the soft tissues overlying the sternocleidomastoid muscle. [Color figure can be viewed at wileyonlinelibrary.com]

**FIGURE 2:**
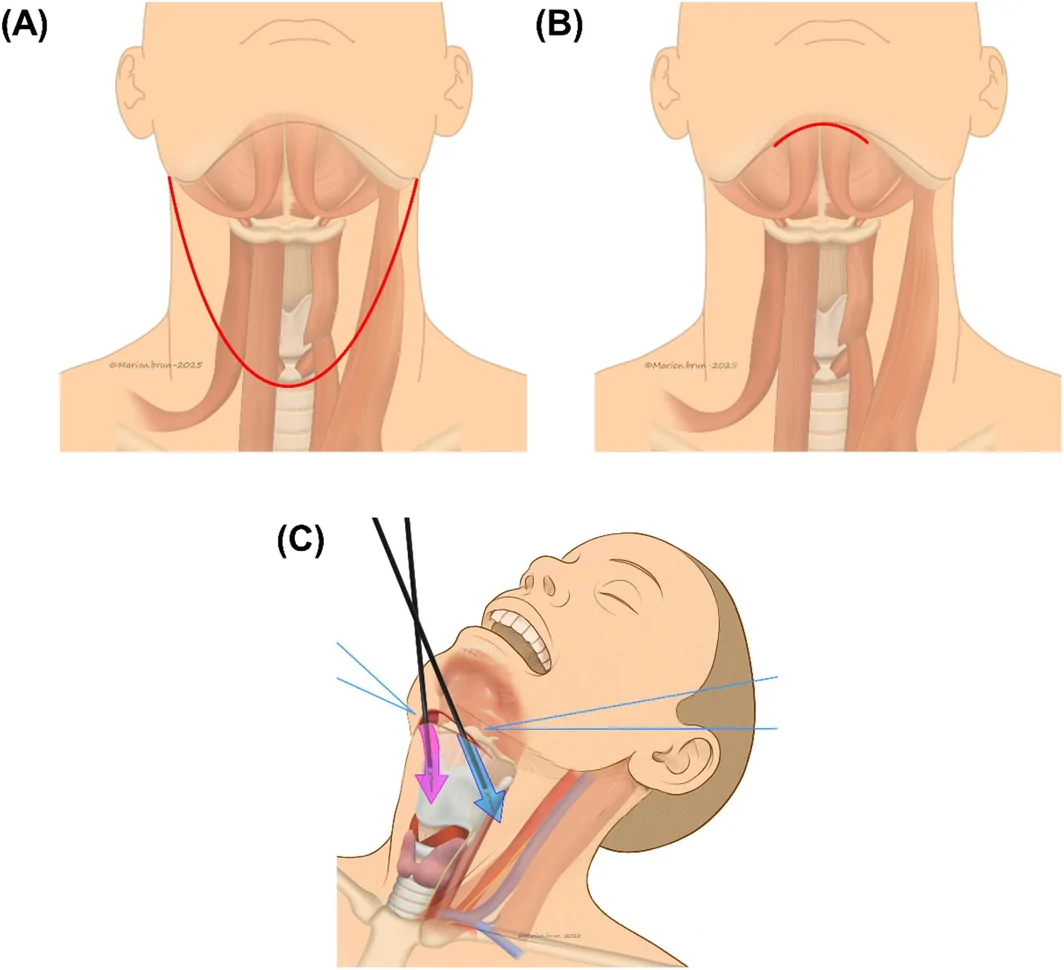
Difference between skin incisions for traditional approach to the bilateral neck dissection (A) and the submental approach robotic‐assisted neck dissection (SARAND) (B). Possible robot docking and working spaces enabling level I to IV bilateral neck dissection (C). [Color figure can be viewed at wileyonlinelibrary.com]

**FIGURE 3:**
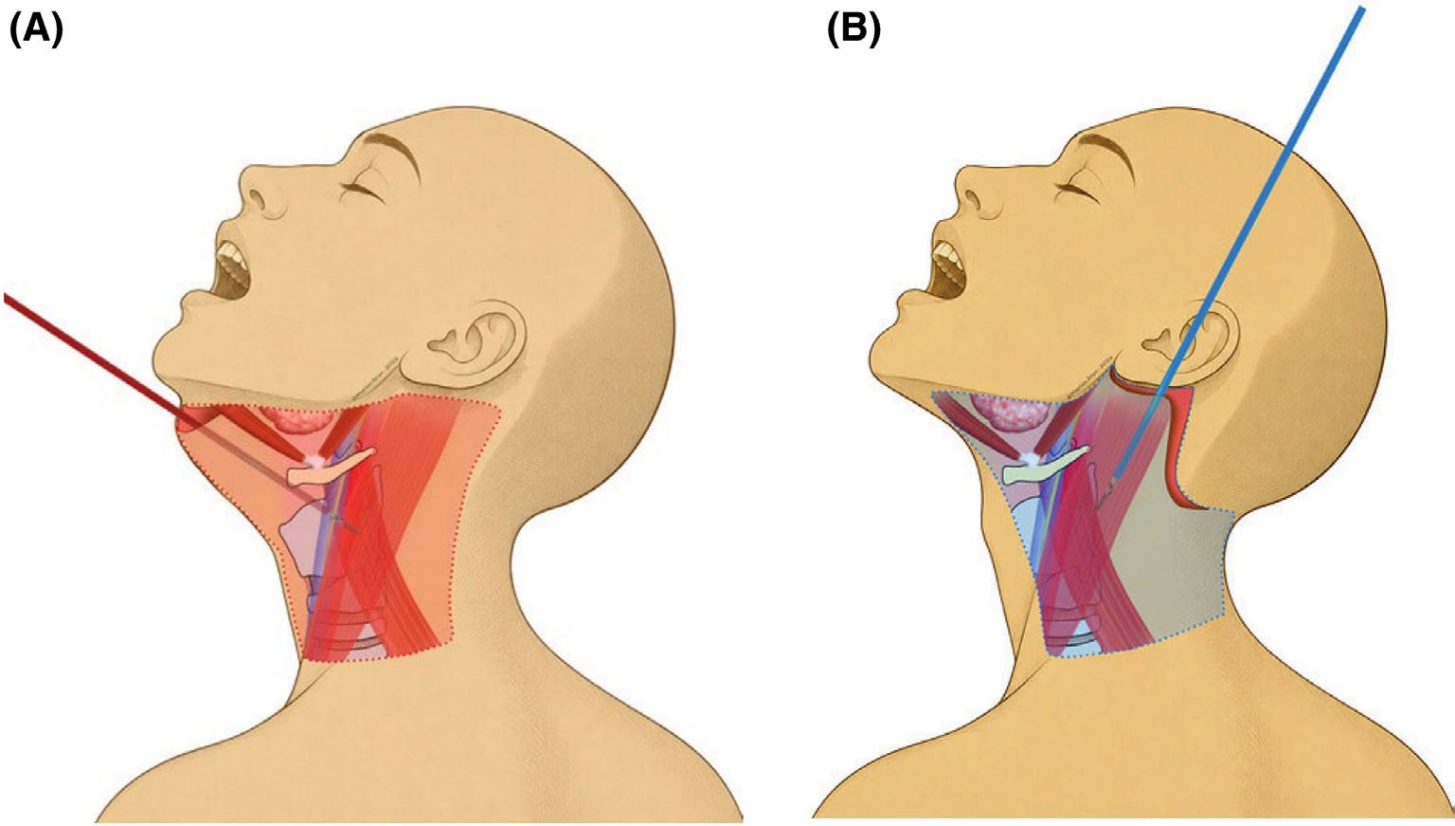
(A) Submental approach robotic‐assisted neck dissection (SARAND): The submental approach provides direct midline access to bilateral cervical lymphatic compartments via a single submental incision, with limited subplatysmal working space. (B) Retroauricular approach: Access to the lateral neck is achieved through a postauricular incision requiring a larger skin‐platysma flap elevation and a longer access route to the cervical lymphatic compartments. [Color figure can be viewed at wileyonlinelibrary.com]

A submental approach robotic‐assisted neck dissection (SARAND) technique is feasible through the RESA‐based approach, minimizing tissue trauma via a small 5–6 cm submental incision, creating a subplatysmal working space above the hyoid bone and thyroid cartilage, and following the investing layer of the deep cervical fascia laterally into the carotid sheath [22, 23, 24] (Figure 1, orange arrow). This technique allows access to both the primary tumor and the neck lymphatics on both sides through a single submental incision and, if needed, a small midline pharyngotomy. It offers an alternative to the classic combination of minimally invasive TORS with the traditional, more invasive trans‐cervical neck dissection. Furthermore, SARAND enables bilateral neck dissection through a single submental entry point, which is particularly advantageous for patients due to the significantly smaller neck incision (Figure 2). A schematic comparison of the working spaces required for SARAND and the retroauricular approach is presented in Figure 3 to highlight the distinct access routes and extent of tissue mobilization.

Although SARAND uses the submental surgical corridor established by the RESA, it is a distinct surgical application differing from the original RESA procedure. Unlike RESA, SARAND does not require resection of the central hyoid bone and is directed primarily toward the lateral cervical compartments to facilitate neck dissection. Furthermore, because this approach can be performed independently of RESA during TORS or other head and neck procedures that require cervical lymphadenectomy (lip, oral cavity, skin, or sinonasal tumor resections), we introduce the acronym SARAND to distinguish this specific neck dissection technique while acknowledging its anatomical foundation within the RESA framework.

This article presents a SARAND cadaveric feasibility study indicating the applicability of both da Vinci Xi and da Vinci SP systems (Intuitive Surgical Inc.) in this procedure.

## 2 Materials and Methods

Three fresh‐frozen human cadavers were utilized to develop the submental approach robotic‐assisted neck dissection (SARAND) in a series of three cadaveric resections conducted between December 2023 and January 2024. The procedures were designed to simulate standard operating room conditions. All procedural steps were photo‐documented and recorded. The laboratory experiments were performed at IRCAD, Strasbourg, France and **“**Medizin im Grünen,” Berlin, Germany. Cadavers were positioned supine with a shoulder roll for better neck extension, and the head maintained in a neutral position. For the multiport dissection using the da Vinci Xi surgical system (Intuitive Surgical Inc.) a subplatysmal working space was established and a Feyh–Kastenbauer retractor was inserted beneath the platysma. The SCM was retracted during the multiport dissection with table‐mounted surgical retractors.

For the single‐port approach, using the da Vinci SP surgical system (Intuitive Surgical Inc.), a similar working space was created after which the da Vinci SP Access Port kit (Intuitive Surgical Inc.) was installed. The following dissection was performed with insufflation at 6 mmHg and a maximum flow rate setting of 40 L/min. Gas insufflation was utilized in the SP approach to maintain a stable subplatysmal working cavity, as the single‐port robotic platform requires a sealed operative space for optimal instrument deployment and visualization. Compared with retractor‐based exposure, insufflation provided uniform elevation of the cervical skin flap, reduced soft tissue collapse, and improved visualization in the narrow cervical workspace.

The article presents experimental use of the da Vinci SP surgical system (Intuitive Surgical Inc.), as its intended use in the EU is limited to transoral procedures.

## 3 Results

SARAND procedures were performed on three fresh‐frozen human cadavers without a previous history of neck surgery—characteristics of the models are available in Table 1.

**TABLE 1:** Characteristics of the cadaveric models.

	Age while deceased	Sex	Height (cm)	Weight (kg)	BMI
1.	75	Male	173	52.6	17.6
2.	98	Female	160	34	13.2
3.	80	Male	162	74	28.2

### 3.1 Multiport SARAND – Cervical Levels I to IV

**FIGURE 4:**
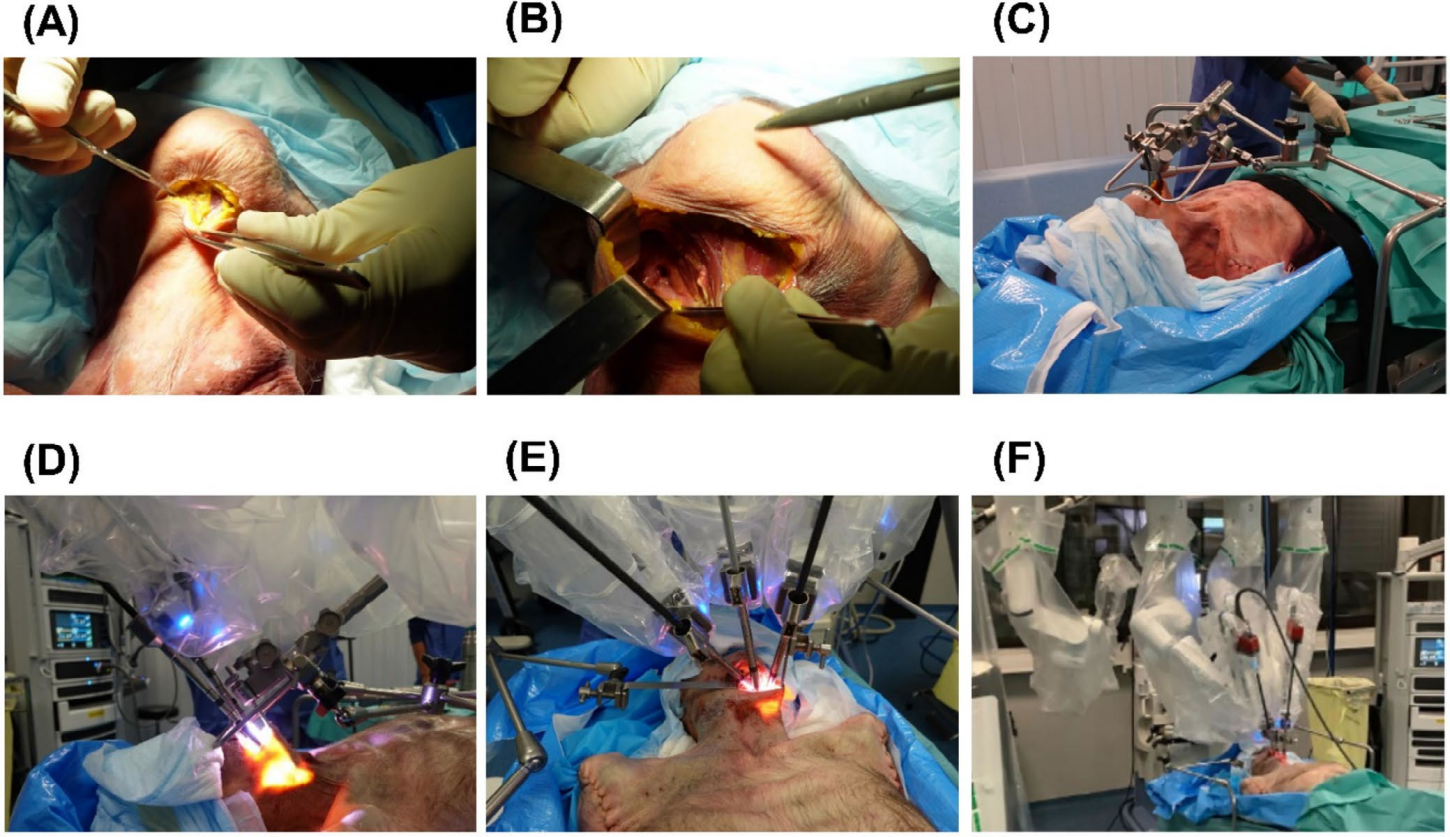
Neck incision, working space creation and docking for the multiport submental approach robotic‐assisted neck dissection (SARAND) to the level I to IV neck dissection. (A) Submental incision; (B) Creation of a “Sistrunk” subplatysmal working space; (C) Retractor position; (D) Docking for level II–IV neck dissection; (E and F) Docking with boom rotated toward head for optimal visualization of I and II levels. [Color figure can be viewed at wileyonlinelibrary.com]

The patient is positioned supine with the head in neutral alignment. A 5–6 cm submental incision is made along the inferior border of the mental region of the mandible (Figure 4A). An 8 × 8 cm subplatysmal working space is created by opening the pretracheal fascia above the hyoid bone and thyroid cartilage, with lateral extension to identify the inferior border of the submandibular gland, the digastric muscle, and the anterior border of the SCM. The SCM is gently lateralized to expose the lymphatic and vascular compartments (Figure 4B). A Feyh‐Kastenbauer retractor is positioned on the mandible and under the platysma to maintain a central working space (Figure 4C). A table‐mounted retractor is then installed on the side of the neck dissection with a blade to retract the SCM and maintain the exposure to the lymphatic and vascular compartment. The multiport robotic system is then docked with a 30‐degree camera positioned in the caudal direction, using Maryland bipolar forceps and monopolar curved scissors, with the fourth arm stowed (Figure 4D).

**FIGURE 5:**
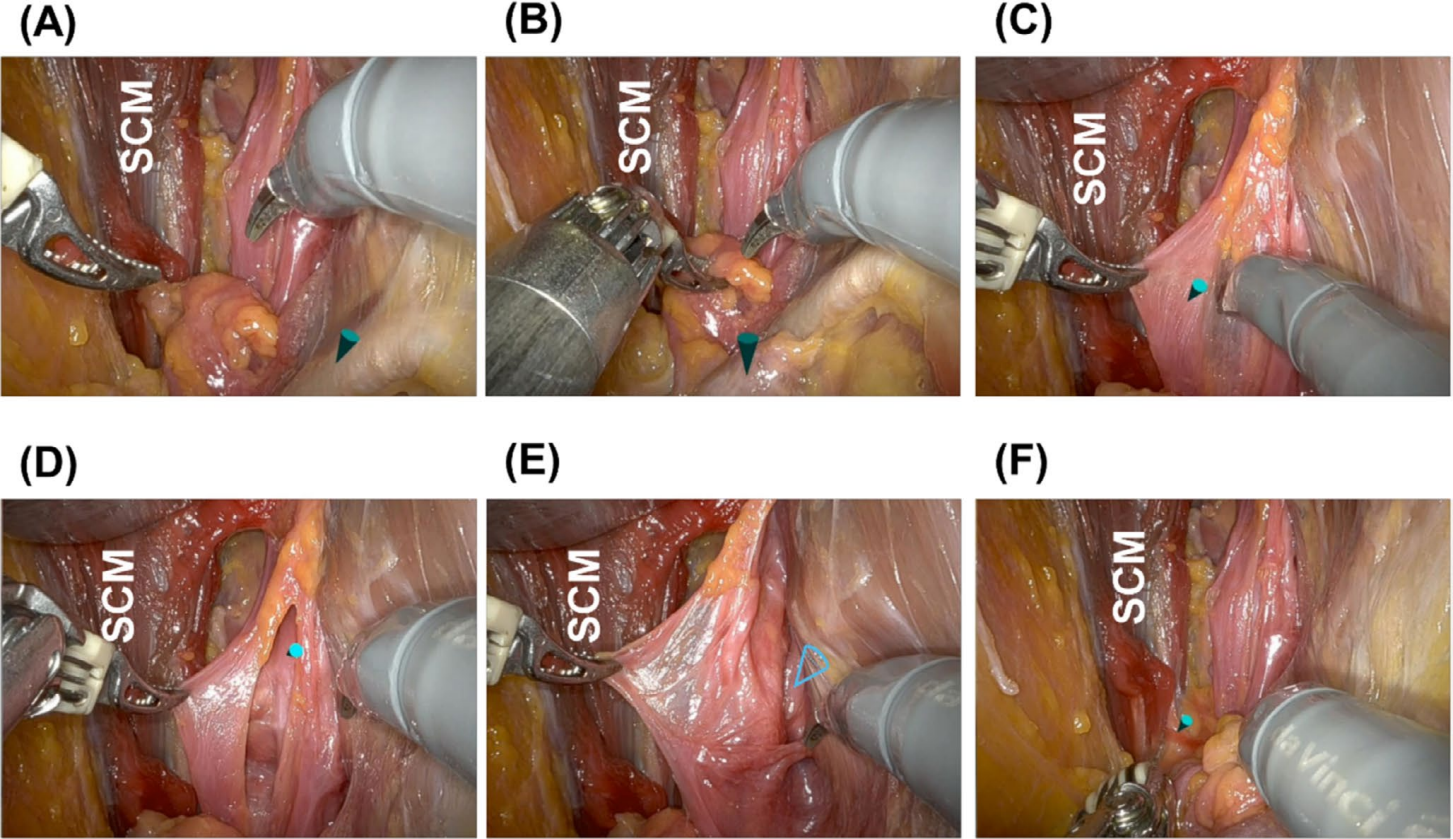
Multiport left‐sided submental approach robotic‐assisted neck dissection (SARAND) (robot boom oriented caudally)—identification of critical structures: (A) Digastric muscle; (B) Submandibular gland; (C) Internal jugular vein; (D) Vagus nerve; (E) Common carotid artery; (F) Accessory nerve. SCM, sternocleidomastoid. [Color figure can be viewed at wileyonlinelibrary.com]

Dissection begins with the identification of the inferior border of the submandibular gland and the digastric muscle (Figure 5A,B). The carotid sheath is then opened to expose the internal jugular vein, vagus nerve, and common carotid artery (Figure 5C–E). This is followed by level III dissection, proceeding toward exposure of the deep cervical fascia, cervical plexus branches, and identification of the omohyoid muscle, followed by level IV dissection (Videos 1 and 2). Next, the accessory nerve is identified (Figure 5F). The robot is then redocked with the boom rotated 180 degrees in the cranial direction (Figure 4E,F), enabling comprehensive dissection of levels Ib, IIa, and IIb. We enclose three videos of the multiport SARAND—Videos [Link], [Link].

### 3.2 Single‐Port SARAND—Cervical Levels I to IV

**FIGURE 6:**
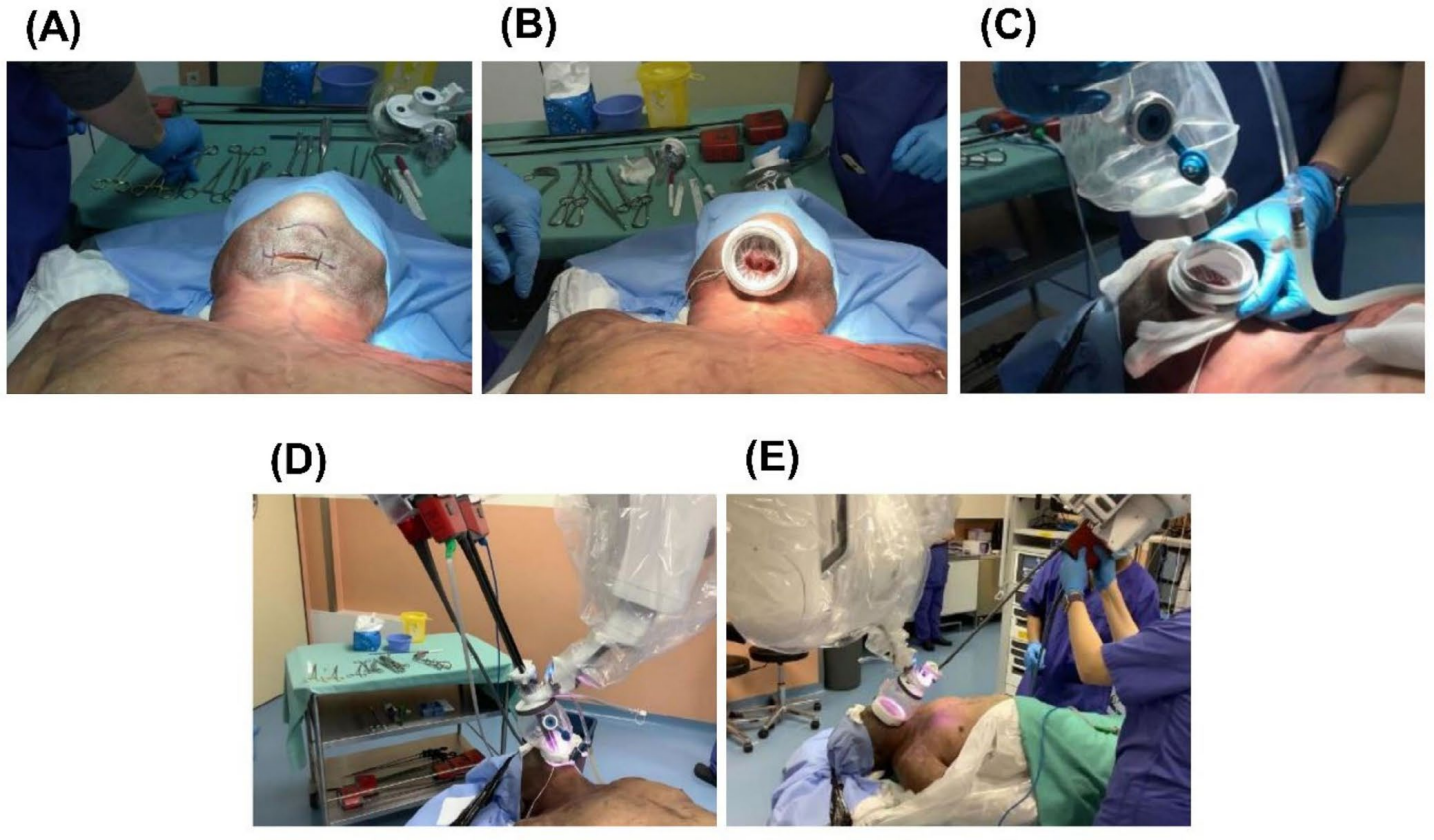
Neck incision, working space creation and docking for the single‐port submental approach robotic‐assisted neck dissection (SARAND) to the level I to IV neck dissection; (A) Submental incision; (B) Wound protector placement; (C) Da Vinci SP Access Port placement; (D) Docking for level II‐IV neck dissection; (E) Docking with boom rotated toward head for optimal visualization of I and II levels. [Color figure can be viewed at wileyonlinelibrary.com]

**FIGURE 7:**
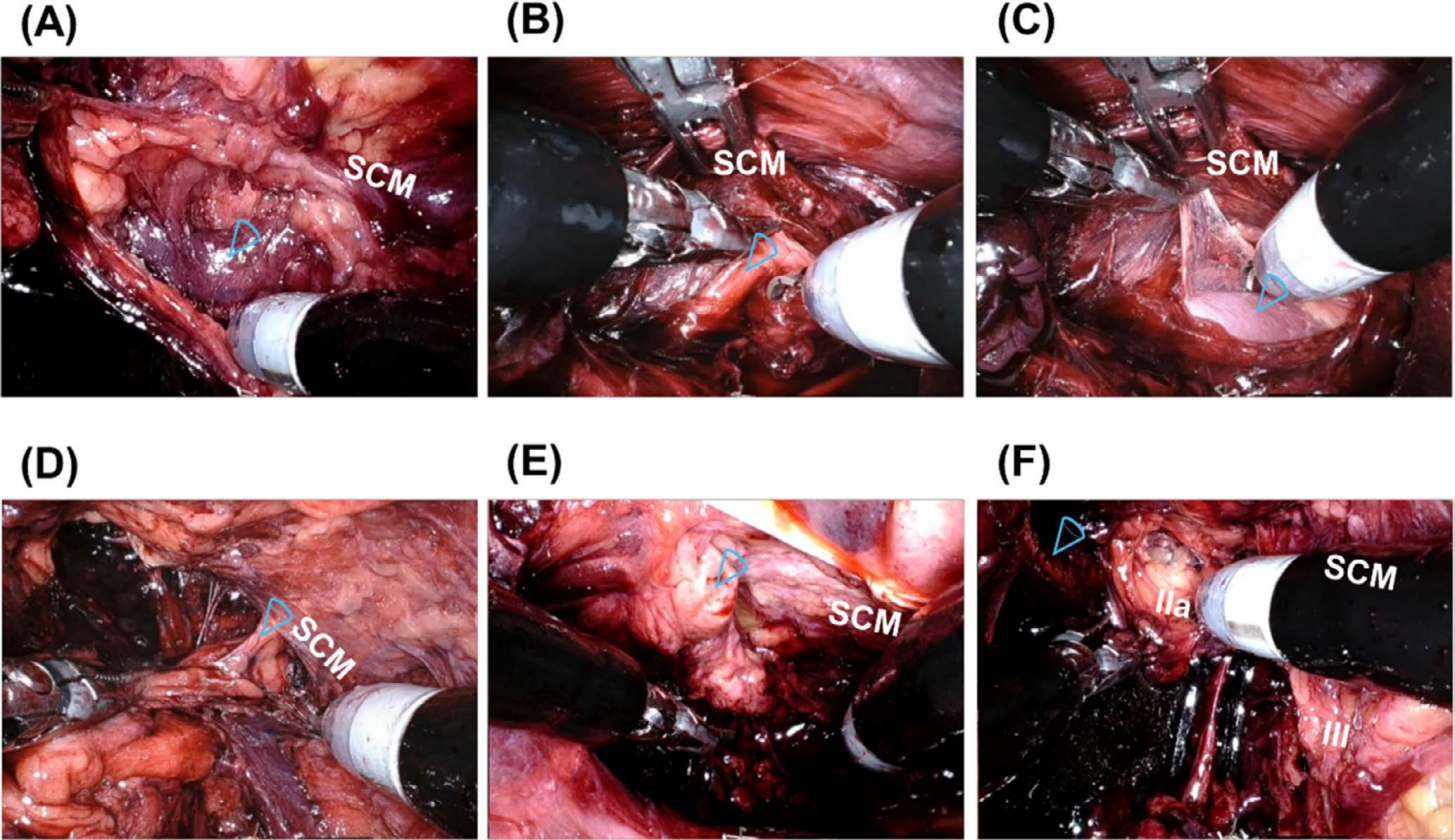
Single‐port left‐sided submental approach robotic‐assisted neck dissection (SARAND) – identification of critical structures: (A) Internal jugular vein; (B) Vagus nerve; (C) Common carotid artery; (D) Accessory nerve; (E) Submandibular gland; (F) Digastric muscle. SCM, sternocleidomastoid, IIa, neck lymph nodes level IIa; III, neck lymph node level III. [Color figure can be viewed at wileyonlinelibrary.com]

The patient is positioned supine with the head in a neutral alignment. Access to the cervical lymphatics is established in the same manner as with the Xi system, using a 5–6 cm single submental incision which is slightly more centered over the hyoid bone (Figure 6A), followed by the creation of a subplatysmal working space. A wound protector (Figure 6B) and the da Vinci SP Access Port (Figure 6C) are then inserted and insufflated to 6 mmHg with a maximum flow rate setting of 40 L/min. The single‐port robotic system is docked in a caudal direction, employing Maryland Bipolar Forceps (Arm 1), Cadiere Forceps (Arm 2), and Monopolar Curved Scissors (Arm 3) (Figure 6D, Figure 7). If necessary, cranial boom rotation provides excellent visualization of levels Ib and IIb (Figure 6E). Neck dissection is performed similarly to the multiport SARAND(Figure 7A–F).

The procedural timing for creating the working space and robot docking for both multiport and single‐port SARAND is provided in Table 2.

**TABLE 2:** Timing of the working space creation and robot docking.

	Cadaver 1 (multiport SARAND)	Cadaver 2 (multiport SARAND)	Cadaver 3 (single‐port SARAND)
Submental incision and subplatysmal working space creation	14 min	21 min	13 min
Robot docking	6 min	6 min	7 min

## 4 Discussion

In this article, we present the surgical technique of a minimally invasive, endoscopic, robotic‐assisted approach to conduct bilateral neck dissections of levels I to IV through a single submental incision—SARAND. It is based on the previously described RESA technique. To demonstrate the feasibility of this approach, we successfully performed three cadaveric bilateral neck dissections using a single submental incision, employing both multiport and single‐port da Vinci robotic systems.

We have previously demonstrated the limitations of the RESA‐based lateral neck dissection in a cadaveric study [22]. The earlier conception required the insertion of vestibular and facelift ports in multiport resection, making the procedure quite invasive, time‐consuming, and focused on one side only—presenting drawbacks similar to those of the retroauricular and transaxillary approaches [26, 27, 28]. Moreover, a dissection of level Ia and Ib was not possible. The novel SARAND technique uses only a single midline submental incision of 5–6 cm; however, it is complemented either with a table‐mounted retractor (multiport approach) or gas insufflation (single port approach), which provides sufficient access for single‐port and multiport instrumentation to bilateral neck dissection. Contrary to the earlier conception, it also allows addressing level Ib and IIb via cranial rotation of the boom for better visualization, depending on individual anatomical conditions.

The most important advantage of SARAND is that it offers favorable access for bilateral neck dissections through one single midline incision. In single‐port SARAND, positioning the incision approximately 1–2 cm below the mandibular border allows for placement of the wound protector's inner ring while keeping the scar cosmetically acceptable. Based on cadaveric experience, the 5–6 cm incision is recommended to allow safe development of the working space. Shorter incisions used in previous attempts restricted visibility and access, potentially increasing the risk of injury to vascular structures. From an aesthetic standpoint, a 5–6 cm scar in the submental region is not expected to be prominently visible due to its favorable location, particularly if placed in the submental crease as done for the multi‐port approach.

From a technical perspective, several factors facilitated the SARAND procedure. Adequate cervical extension and meticulous development of a wide subplatysmal working space were critical for exposure and instrument mobility. In the single‐port approach, stable insufflation improved maintenance of the operative cavity and instrument triangulation, whereas in the multiport approach, lateral retraction of the SCM was essential for exposure of levels III and IV. Furthermore, cranial rotation of the robotic boom facilitated access to levels Ib and IIb and improved visualization of the superior neck.

Although exposure of levels I–IV, including level IIb, was feasible using both multiport and single‐port systems, anatomical variability may limit the clinical applicability of SARAND. Exposure of the inferior neck, particularly level IV, may become more challenging in patients with short or obese necks due to increased soft tissue volume, limited cervical extension, and less favorable instrument angulation. Similarly, bulky nodal disease, extranodal extension, prior neck surgery, or post‐radiotherapy fibrosis may limit the creation of working space and visualization. Therefore, early clinical application of SARAND should likely focus on carefully selected patients with favorable cervical anatomy and limited nodal involvement.

Notably, SARAND provides the opportunity for minimally invasive surgical treatment of both primary tumor—via medial pharyngotomy [22, 23]—and bilateral neck dissection through the same submental incision. This integrated approach may potentially offer reduced neck morbidity, improved cosmetic outcomes, and decreased operating time compared to other robotic‐assisted approaches by utilizing a shared working space.

Outcomes of robotic‐assisted neck dissection are equivalent to the traditional approach in terms of nodal yield, complication rate, postoperative recovery, and recurrence, according to two meta‐analyses [27, 28]. Both studies reported improved cosmetic results with robotic techniques, although these procedures required longer operative times. Lee et al. found that traditional neck dissection carried a lower risk of marginal mandibular nerve injury [27]. However, these analyses included studies utilizing only retroauricular, transaxillary, and modified facelift robotic approaches. As RESA and SARAND are novel techniques, clinical data will have to be generated [22, 23, 24].

Compared to other robotic‐assisted approaches, SARAND appears to be less invasive, offering a potentially lower morbidity profile while maintaining the favorable cosmetic outcomes. Notably, in bilateral neck dissection, operative time may be reduced due to the single, midline incision, which provides access to cervical lymphatics on both sides. Moreover, direct access of the vascular and lymphatic compartment from the midline helps to avoid injuries to the greater auricular nerve, thus preserving sensation of the skin. These anticipated conceptual advantages remain hypothetical in the absence of clinical data, and further in vivo studies are required to validate the feasibility, safety, and functional outcomes of SARAND before considering its integration into routine surgical practice.

A clinical validation of SARAND is planned by the authors in a feasibility trial, assessing lymph node yield (LNY), procedure‐related complications, postoperative recovery, functional and aesthetic outcomes. Initial candidates for the SARAND trial are expected to be carefully selected patients with limited cervical nodal burden, including cN0 and cN1 disease, requiring unilateral or bilateral neck dissection. The anticipated complication profile is expected to be comparable to that reported for traditional and other robotic‐assisted neck dissection techniques [27, 28]. They include perioperative bleeding, cervical emphysema, infection, and cranial nerve injury, particularly involving the marginal mandibular and spinal accessory nerves.

In summary, we present in this cadaveric study a minimally invasive approach to bilateral neck dissections of level I to IV. Potential advantages of the SARAND technique include concealed scarring, reduced operative time, and preserved skin sensation. Furthermore, by preserving subcutaneous lymphatic structures, SARAND may reduce the risk of postoperative lymphedema and support better functional outcomes related to swallowing and respiration, resulting in shorter recovery and enhanced quality of life. Finally, SARAND enables minimally invasive resection of the primary tumor and bilateral neck dissection through the same 5–6 cm submental incision.

## Funding

This work was supported by Intuitive Surgical Inc.

## Ethics Statement

This study was performed on three cadavers. The individuals had signed a consent form prior to their deaths, authorizing the use of their bodies for research. The study was performed in accordance with ethical standards and the 1964 Declaration of Helsinki and its subsequent updates. Institutional review board approval was not required for this study because it did not involve living human subjects.

## Conflicts of Interest

Ka Hei Suen and Markus Rheinwald are employees and own stock in Intuitive Surgical Inc.

## Supporting information


**Video 1** Left neck dissection: levels I to IV.

**Video 2** Right neck dissection: levels I to IV.

**Video 3** Left neck dissection: level Ib.

**Data S1:** Videos Summary.

## Data Availability

Data sharing not applicable to this article as no datasets were generated or analysed during the current study.
