# Supplementary material for: Submental Approach Robotic‐Assisted Neck Dissection (SARAND) for Minimally Invasive Bilateral Neck Dissection: A Feasibility Cadaver Dissection Study
Source: Head Neck. 2026 Jul 14;48(10):2934–42. doi: 10.1002/hed.70397 (PMC13595487; doi:10.1002/hed.70397)
Supplement: Supplementary file 1 [file HED-48-2934-s002.docx]

Each dissection demonstrated in the videos is preceded by preparation of the surgical working space. The patient is positioned supine with the head in neutral alignment. A 5-6 cm submental incision is made along the inferior border of the mental region of the mandible. An 8 x 8 cm subplatysmal working space is created by opening the pretracheal fascia above the hyoid bone and thyroid cartilage, with lateral extension to identify the inferior border of the submandibular gland, the digastric muscle, and the anterior border of the SCM. The SCM is gently lateralized to expose the lymphatic and vascular compartments. A Feyh-Kastenbauer retractor is positioned on the mandible and under the platysma to maintain a central working space. A table mounted retractor is then installed on the side of the neck dissection with a blade to retract the SCM and maintain the exposure to the lymphatic and vascular compartment. The multiport robotic system is then docked with a 30-degree camera positioned in the caudal direction, using Maryland bipolar forceps and monopolar curved scissors, with the fourth arm stowed.

Video 1. Left neck dissection: levels I to IV.

The dissection begins with identification of the left carotid sheath, including internal jugular vein and common carotid artery. Levels IV and III are then dissected in a caudal-to-cranial direction. The spinal accessory nerve is subsequently identified, followed by dissection of levels IIa and IIb with exposure of the facial artery. To optimize visualization of level IIb, the robot is redocked and the boom is rotated, allowing completion of this dissection. Finally, level Ib is addressed with dissection from the mylohyoid muscle, during which the submandibular duct and facial artery are clearly identified.

Video 2. Right neck dissection: levels I to IV.

The dissection begins with identification of the right carotid sheath, including the internal jugular vein. Levels IV and III are dissected in a caudal-to-cranial direction, followed by dissection of levels IIa and IIb with identification of the spinal accessory nerve. Dissection of level Ib is then initiated with exposure of the facial artery. To optimize visualization of level Ib, the robot is redocked and the boom is rotated, allowing completion of the dissection.

Video 3. Left neck dissection: level Ib.

The dissection begins with identification of the mylohyoid muscle and its vascular pedicle. The submandibular duct is then identified, and the dissection is gradually completed.
